# MicroRNA-153/Nrf-2/GPx1 pathway regulates radiosensitivity and stemness of glioma stem cells via reactive oxygen species

**DOI:** 10.18632/oncotarget.4292

**Published:** 2015-06-18

**Authors:** Wei Yang, Yueming Shen, Jing Wei, Fenju Liu

**Affiliations:** ^1^ Department of Radiobiology, School of Radiological Medicine and Protection, Medical College of Soochow University, Collaborative Innovation Center of Radiation Medicine of Jiangsu Higher Education Institutions, Soochow University, Suzhou, Jiangsu 215123, China

**Keywords:** microRNA-153, Nrf-2, GPx1, radiosensitivity, glioma stem cells

## Abstract

Glioma stem cells (GSCs) exhibit stem cell properties and high resistance to radiotherapy. The main aim of our study was to determine the roles of ROS in radioresistance and stemness of GSCs. We found that microRNA (miR)-153 was down-regulated and its target gene nuclear factor-erythroid 2-related factor-2 (Nrf-2) was up-regulated in GSCs compared with that of non-GSCs glioma cells. The enhanced Nrf-2 expression increased glutathione peroxidase 1 (GPx1) transcription and decreased ROS level leading to radioresistance of GSCs. MiR-153 overexpression resulted in increased ROS production and radiosensitization of GSCs. Moreover, miR-153 overexpression led to decreased neurosphere formation capacity and stem cell marker expression, and induced differentiation through ROS-mediated activation of p38 MAPK in GSCs. Nrf-2 overexpression rescued the decreased stemness and radioresistance resulting from miR-153 overexpression in GSCs. In addition, miR-153 overexpression reduced tumorigenic capacity of GSCs and increased survival in mice bearing human GSCs. These findings demonstrated that miR-153 overexpression decreased radioresistance and stemness of GSCs through targeting Nrf-2/GPx1/ROS pathway.

## INTRODUCTION

A growing body of evidence now suggests that a stem-like subpopulation exist in many tumor types, including glioma [1, 2]. These small population of cells with self-renewal capacity and immature phenotype in glioma, called “glioma stem cells” (GSCs), which exhibit stem cell properties and have ability to initiate and propagate tumors [3, 4]. GSCs are also characterized by high resistance to chemotherapy and radiotherapy [5–7]. To date, only a few molecular mechanisms for GSCs resistance to apoptotic signals have been identified. It was reported that GSCs displayed resistance to radiation due to elevated activation of the DNA damage checkpoint and DNA repair [5], whereas chemoresistance of GSCs was linked to increased drug efflux caused by the ABCG2 transporter [8]. Elucidation of the molecular mechanisms underlying the therapeutic resistance of GSCs would contribute to identification of novel targets of therapeutic intervention and prolongation of patient survival.

Reactive oxygen species (ROS), byproducts of aerobic metabolism, play pivotal roles in tumorigenesis, metastasis, and therapeutic resistance of cancer [9]. However, the roles of ROS in cancer stem cells (CSCs), including GSCs, remain poorly understood [10, 11]. Although previous studies demonstrated that breast cancer stem cells contained lower ROS levels and enhanced ROS defenses compared to their non-tumorigenic progeny, which might contribute to tumor radioresistance, little is known about the biological effects of ROS and their regulating mechanisms in GSCs [12].

Nuclear factor-erythroid 2-related factor-2 (Nrf-2/NFE2L-2), a redox sensitive transcription factor, regulates the expression of several detoxifying enzymes, such as glutathione transferases (GSTs), glutathione peroxidase (GPx), catalase, and nicotinamide adenine dinucleotide phosphate (reduced) (NADPH): quinone oxidoreductase-1 (NQO-1), through binding to antioxidant response element (ARE) within gene promoters [13]. Nrf-2 function is regulated post-translationally by its negative regulator Kelch-like ECH-associated protein 1 (Keap1) that binds Nrf-2 and induces cytoplasmic Nrf-2 degradation [14]. Nrf-2 is also transcriptionally regulated by arylhydrocarbon receptor (AhR) [15, 16]. In addition, studies showed that several microRNAs (miRs) could target the 3′ UTR of Nrf-2 mRNA and inhibit Nrf-2 expression in neuronal or non-neuronal models [17–19]. However, the post-transcriptional regulation of Nrf-2 by miRs in GSCs and glioma cells remain elusive.

In this study, we investigated the role of ROS in radioresistance and stemness of GSCs and the regulating mechanisms of ROS in GSCs. Our results showed that Nrf-2 was a target gene of miR-153 in GSCs and low level of miR-153 rescued Nrf-2 expression leading to activation of GPx1 transcription and decreased ROS level, which contributed to radioresistance of GSCs. Moreover, we found that miR-153 overexpression resulted in radiosensitization of GSCs as well as decreased stemness and increased differentiation through ROS-mediated activation of p38 MAPK in GSCs. Nrf-2 overexpression rescued the decreased stemness and radioresistance resulting from miR-153 overexpression in GSCs. In addition, miR-153 overexpression reduced tumorigenic capacity of GSCs and increased survival in mice bearing human GSCs.

The pivotal role of the microRNA-153/Nrf-2/GPx1 pathway in the control of radioresistance and stemness of GSCs demonstrated in this study suggested that the pathway might be a novel therapeutic target of GSCs.

## RESULTS

### Intrinsic radiosensitivity and ROS formation of GSCs and non-GSCs glioma cells

**Figure 1:**
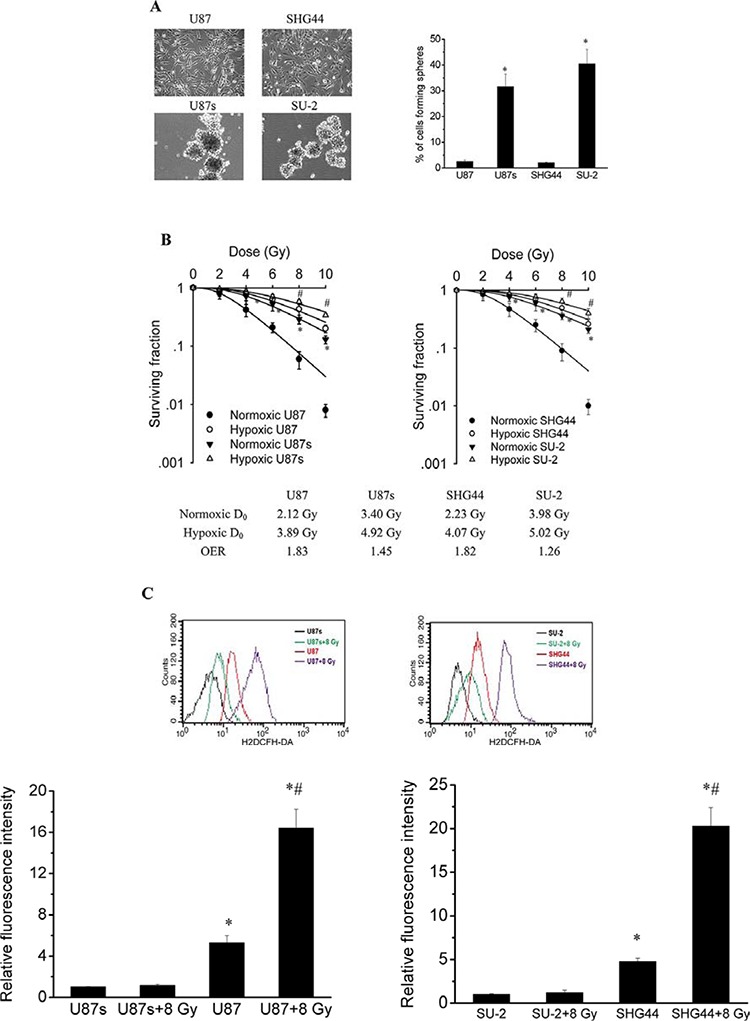
GSCs were more radioresistent and generated less ROS than non-GSCs glioma cells **A.** The percentage of cells plated at 100 cells per well in 96-well plates forming neurospheres. *P < 0.01 vs U87 or SHG44. **B.** Survival curves of GSCs and non-GSCs glioma cells. After exposure to 0, 2, 4, 6, 8 and 10 Gy X-ray irradiation under normoxia or hypoxic conditions, cell survival fractions were examined and the survival curves of cells were obtained from data fitting according to the linear quadratic model. Error bars indicate the standard error of the mean of three individual experiments. *P < 0.01 vs normoxic U87 or SHG44. ^#^*P* < 0.05 vs hypoxic U87 or SHG44. OER (oxygen enhancement ratio) was calculated as the ratio of hypoxic D_0_ (mean lethal dose) to normoxic D_0_. D_0_ is the dose required to reduce the fraction of surviving cells to 37% of its previous value. **C.** Flow cytometric analysis of ROS formation using the H2DCFH-DA probe in GSCs and non-GSCs glioma cells after exposure to ionizing radiation. *P < 0.01 vs U87s or SU-2. ^#^*P* < 0.01 vs U87 or SHG44.

To test the self-renewal capabilities of GSCs and non-GSCs glioma cells, cells were cultured in serum-free medium to generate neurospheres. Neurosphere formation assay showed that neurosphere formation rate of GSCs was significantly higher than that of non-GSCs glioma cells, as shown in Figure 1A. After exposure to 0, 2, 4, 6, 8 and 10 Gy X-ray irradiation under normoxia (atmospheric conditions, 21% O_2_) or hypoxic conditions (24 h, 1% O_2_), cell survival fractions were examined and the survival curves of cells, as shown in Figure 1B, were obtained from data fitting according to the linear quadratic model. It is clear that GSCs, U87s and SU-2, were more radioresistent than glioma cell lines, U87 and SHG44, respectively, under normoxia or hypoxic conditions. In addition, the GSCs showed a slightly lower OER than the non-GSCs glioma cells. One mechanism behind this radioresistance and decreased OER might be a drastically reduced amount of free radicals formed following irradiation, which could reduce the number of DNA double strand breaks, so we next tested whether ROS formation in irradiated GSCs were repressed. ROS formation was examined using cells loaded with H2DCFH-DA. The basal ROS formation in GSCs was significantly reduced compared with that of non-GSCs glioma cells. ROS generation was significantly increased in non-GSCs glioma cells, whereas ROS generation in GSCs showed no obvious change 1 h after 8 Gy X-ray irradiation, as shown in Figure 1C.

### Redox enzymes expression and activity in GSCs and non-GSCs glioma cells

**Figure 2:**
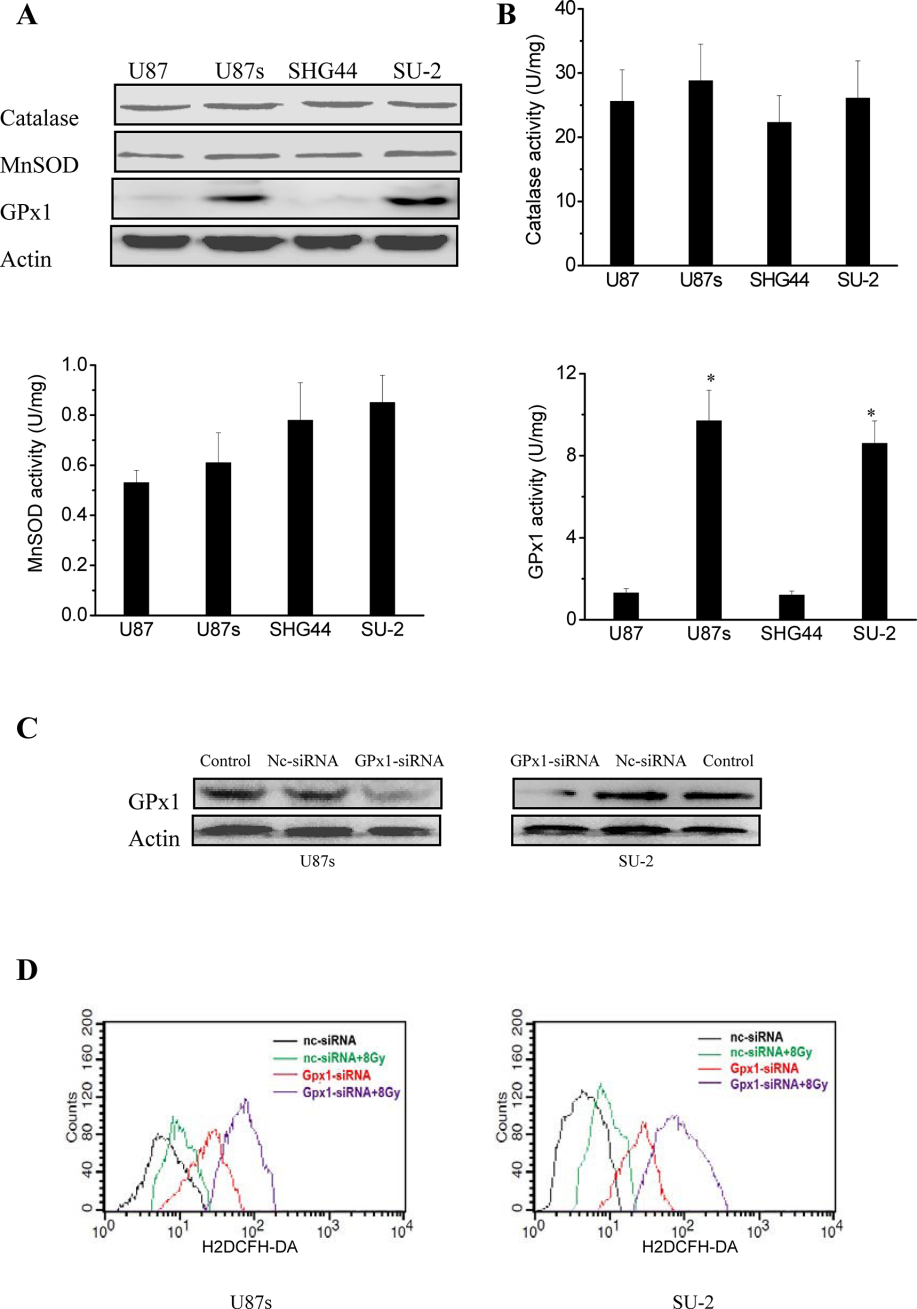

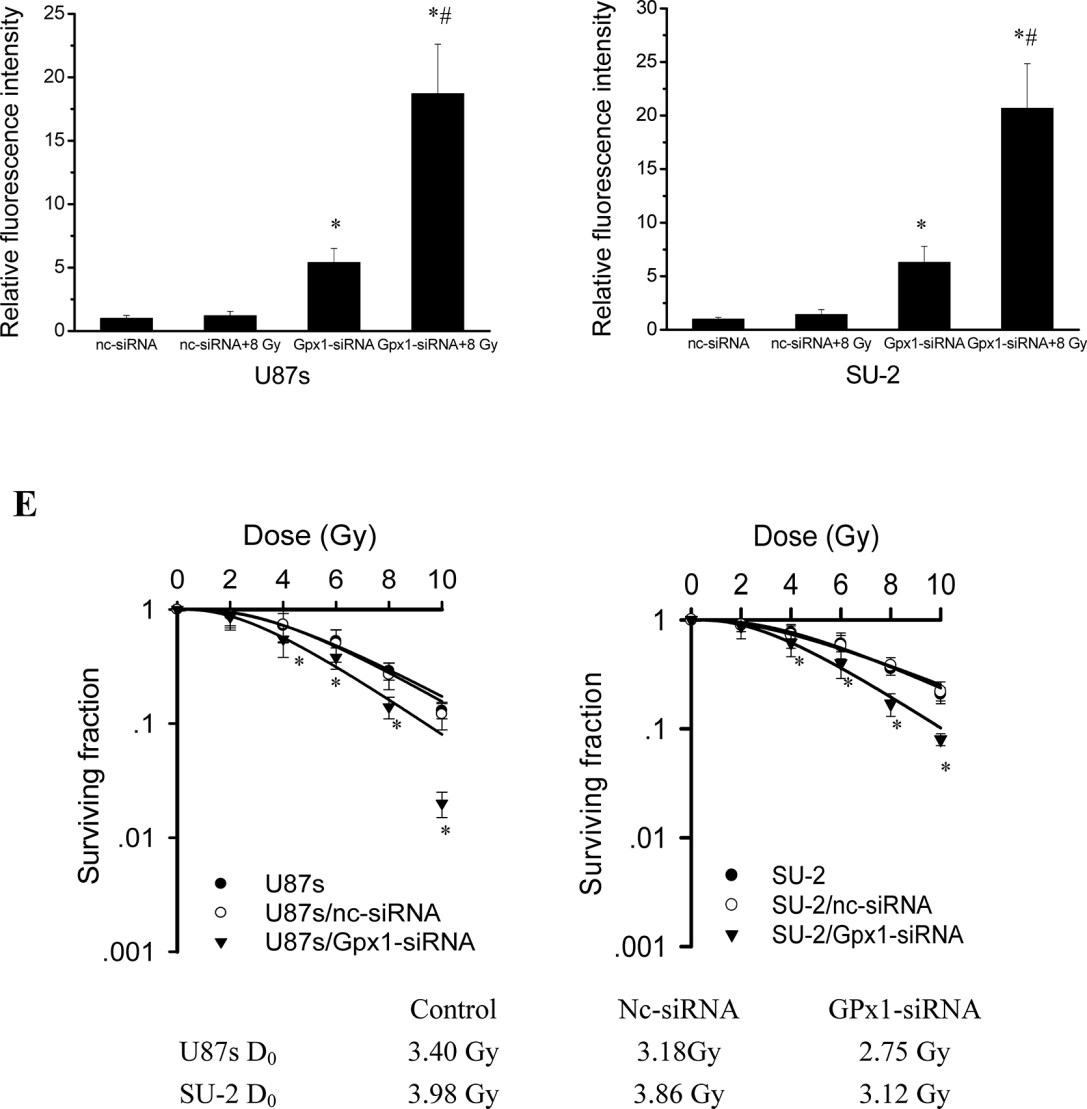
Redox enzymes expression and activity in GSCs and non-GSCs glioma cells **A.** Detection of protein expression of catalase, MnSOD and GPx1 by Western blot. **B.** Enzymatic activities of catalase, MnSOD and GPx1 in GSCs and non-GSCs glioma cells. *P < 0.01 vs glioma cells. **C.** Detection of protein expression of GPx1 in GSCs transfected with GPx1-siRNA by Western blot. **D.** Flow cytometric analysis of ROS formation using the H2DCFH-DA probe in GSCs transfected with GPx1-siRNA. *P < 0.01 vs U87s/nc-siRNA or SU-2/nc-siRNA. ^#^*P* < 0.01 vs U87s/GPx1-siRNA or SU-2/GPx1-siRNA. **E.** Survival curves of GSCs transfected with GPx1-siRNA. After exposure to 0, 2, 4, 6, 8 and 10 Gy X-ray irradiation, cell survival fractions were examined and the survival curves of cells were obtained from data fitting according to the linear quadratic model. Error bars indicate the standard error of the mean of three individual experiments. *P < 0.01 vs U87s or SU-2. D_0_ (mean lethal dose) was calculated by the linear quadratic model.

The differences in the radiosensitivity and ROS formation observed between GSCs and non-GSCs glioma cells led us to postulate differences in their redox-maintaining mechanisms. We therefore tested the protein expression of catalase, MnSOD, CuZnSOD and GPx1 by Western blot, which are the most important enzymes that regulate superoxide and hydrogen peroxide levels in cells. As shown in Figure 2A, catalase and MnSOD were constitutively expressed in all tested GSCs and non-GSCs glioma cells. CuZnSOD was hardly detectable in any tested cells (data not shown). GPx1 protein expression in non-GSCs glioma cells was significantly reduced compared with that of GSCs. We next investigated the enzymatic activities of catalase, MnSOD and GPx1 in GSCs and non-GSCs glioma cells. All the tested cells had very similar levels of basal catalase activity. MnSOD activity between GSCs and non-GSCs glioma cells showed no obvious change. Assays for GPx1 activity showed that non-GSCs glioma cells displayed significantly lower basal GPx1 activity than GSCs (Figure 2B). Moreover, we investigated whether GPx1 downregulation could increase ROS formation and radiosensitize GSCs. After GPx1-siRNA transfection, GPx1 protein expression of GSCs were significantly decreased (Figure 2C), and ROS formation were significantly increased (Figure 2D). Radiosensitivity of GSCs was examined by clonogenic assay 48 h after GPx1-siRNA transfection. As shown in Figure 2E, it is clear that GSCs transfected with GPx1-siRNA were more radiosensitive than GSCs transfected with nc-siRNA. The results indicated that GPx1 downregulation could increase ROS formation and radiosensitize GSCs. Collectively, these data suggest that GPx1 expression contribute to radioresistance of GSCs.

### Regulation mechanisms of GPx1 expression

**Figure 3:**
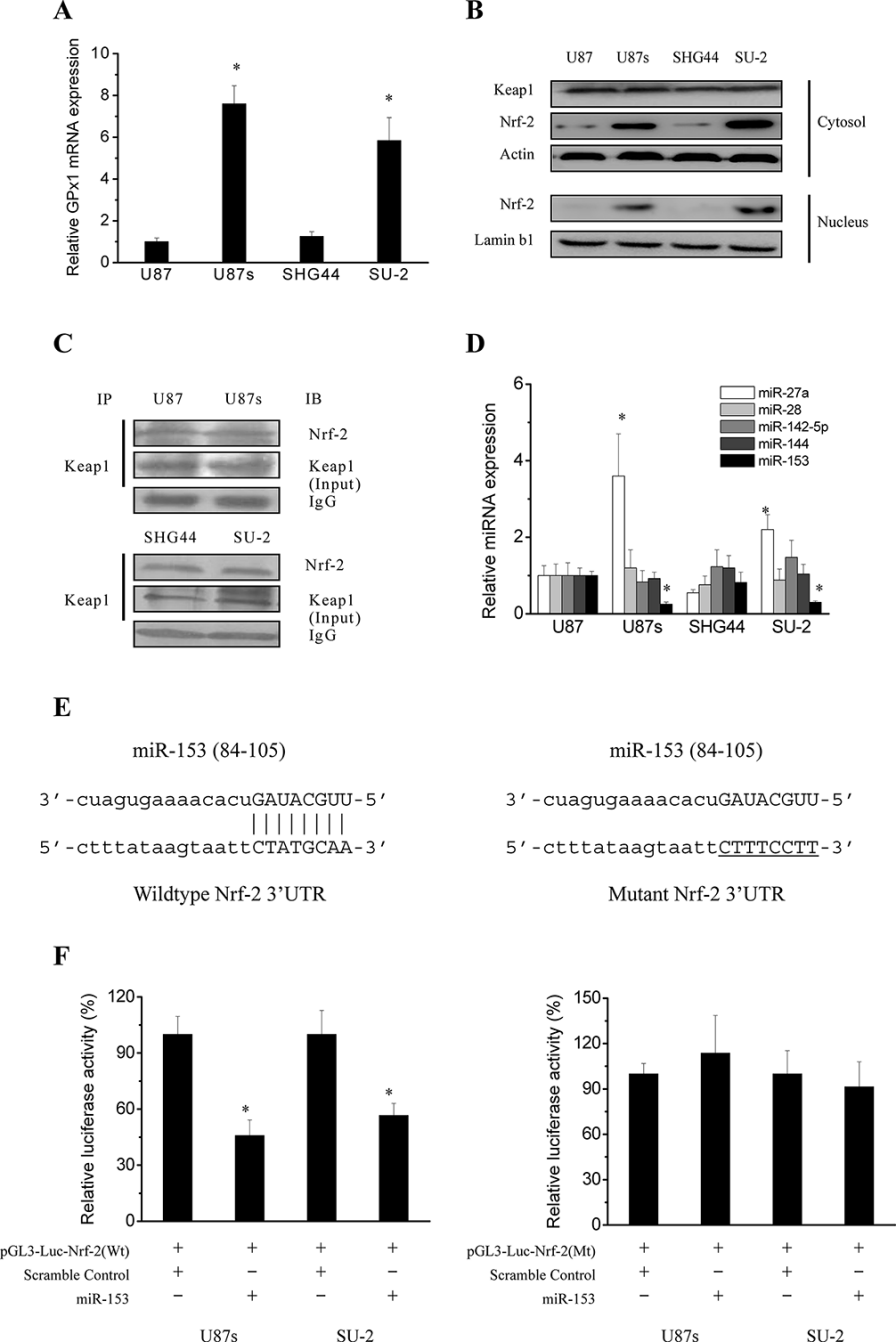
Regulation mechanisms of GPx1 expression **A.** Real-time RT-PCR analysis of GPx1 mRNA expression in GSCs and non-GSCs glioma cells. *P < 0.01 vs non-GSCs glioma cells. **B.** Detection of protein expression of Nrf-2 and Keap1 by Western blot. **C.** Immunoprecipitation analysis of interaction between Nrf-2 and Keap1. Cells were immunoprecipitated with Keap1 and immunoblotted with Nrf-2 antibody. **D.** Real-time RT-PCR analysis of the expression levels of the putative miRs that could target Nrf-2. *P < 0.01 vs U87 cells. **E.** A putative miR-153 target site in the wild type and mutated 3′UTR of Nrf-2. **F.** Relative luciferase activity in GSCs transfected with pGL3-Luc vector containing wild type and mutated 3′UTR of Nrf-2. *P < 0.01 vs cells transfected with scramble oligos.

To gain insights in the possible mechanisms involved in the regulation of GPx1 expression, we first analyzed GPx1 mRNA expression in GSCs and non-GSCs glioma cells. As shown in Figure 3A, Real-time RT-PCR analysis indicates that non-GSCs glioma cells displayed significantly lower GPx1 mRNA levels than GSCs, which corresponded to that of protein expression and enzymatic activity. It is well documented that Nrf-2, a redox-sensitive transcription factor control the transcription of both constitutive and inducible ARE-related genes such as γ-glutamylcysteine synthetase (γ-GCS), heme oxygenase-1 (HO-1) and GPx [13]. Keap1 is a regulator of Nrf-2 by serving as a substrate adaptor for Cullin3-dependent E3 ubiquitin ligase [14]. Therefore, we next detected the Nrf-2 protein expression in cytosol and nucleus of GSCs and non-GSCs glioma cells. As shown in Figure 3B, Nrf-2 protein expression was significantly increased in cytosol and nucleus of GSCs compared with that of non-GSCs glioma cells. Keap1 protein expression in cytosol between GSCs and non-GSCs glioma cells showed no obvious change. As mentioned above, Nrf-2 can be degraded in the cytoplasm through ubiquitination resulting from Keap1/Nrf-2 interaction. In order to examine the possibility that changes in Nrf-2 protein expression in GSCs and non-GSCs glioma cells might be due to differences in Keap1/Nrf-2 interaction, co-immunoprecipitation was performed to examine Keap1/Nrf-2 interaction status. As shown in Figure 3C, When cells were immunoprecipitated with Keap1 and immunoblotted with Nrf-2 antibody, no obvious change was observed in Keap1/Nrf-2 interaction in GSCs and corresponding non-GSCs glioma cells. In addition to post-translational regulation of Nrf-2 expression by Keap1, Nrf-2 is subject to transcriptional and translational regulation. The post-transcriptional regulation of Nrf-2 which occurs chiefly via microRNAs (miRs) is poorly studied in GSCs and non-GSCs glioma cells. Since the 3′ UTR of Nrf-2 transcript is 428 bp in length, many potential miRNA binding sites are possible. Human Nrf-2 3′ UTR was bio-informaticly analysised for miRNA seed sequences using the predicted targets component of “miRecords”, which integrates data from 11 miRNA target prediction programs. Four common miRs (miR-144, 153, 27a and 142–5p) listed in at least 6 individual databases in miRecords were selected. In addition, Nrf-2 has been recently demonstrated to be regulated by miR-28 in non-neuronal models, and miR-144, 153, 27a and 142–5p in neurons [17–19]. To determine potential miRNAs which regulate Nrf-2 expression in GSCs and non-GSCs glioma cells, we first examined the expression level of the above mentioned miRs by real-time PCR. As shown in Figure 3D, endogenous level of miR-27a was significantly increased in GSCs compared with that of non-GSCs glioma cells. Endogenous level of miR-28, 142–5p and 144 showed no obvious change between GSCs and non-GSCs glioma cells. Endogenous level of miR-153 was significantly decreased in GSCs compared with that of non-GSCs glioma cells. As miRs are believed to inversely control mRNA translation, miR-153 were selected as a candidate miR targeting Nrf-2 and we speculated that reduced miR-153 might lead to the enhanced Nrf-2 expression in GSCs. Generally, miRs bind to a 7 to 8 nucleotide match sequences located in the 3′ untranslated region (3′-UTR) of their target mRNA and control their expression [20]. To experimentally determine whether miR-153 directly targets Nrf-2 by binding to its 3′ UTR sequence (Figure 3E), we employed luciferase reporter assays. Typically, a decreased luminescence output in this assay indicated that miRNAs effectively bound to and targeted the 3′ UTR. As shown in Figure 3F, after miR-153 oligos were transfected into GSCs with the wild type reporter construct pGL3-Luc-Nrf-2, luciferase activity was significantly repressed compared with transfection of scramble oligos, whereas mutations in predicted target site of 3′-UTR of Nrf-2 gene abrogated the inhibition by miR-153 oligos. These results suggest that Nrf-2 is a direct and robust target gene of miR-153 in GSCs. Collectively, these data demonstrated that miR-153 expression was down-regulated in GSCs compared with that of non-GSCs glioma cells, which contributed to enhanced Nrf-2 expression resulting in activation of GPx1 transcription.

### MiR-153 overexpression suppressed Nrf-2 expression and Redox enzymes activity in GSCs

**Figure 4:**
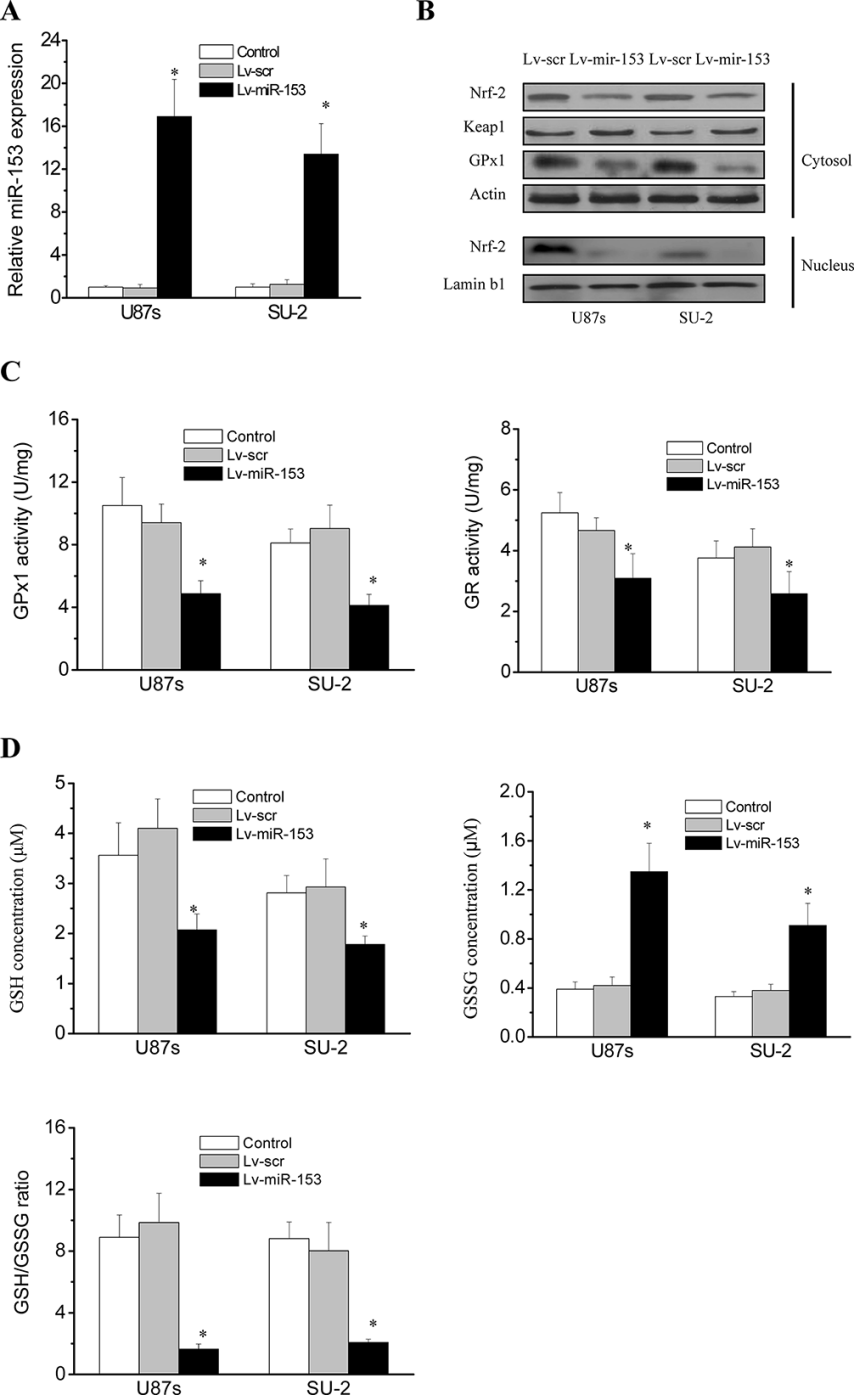
MiR-153 overexpression suppressed Nrf-2 expression and Redox enzymes activity in GSCs **A.** Real-time RT-PCR analysis of miR-153 expression in GSCs. *P < 0.01 vs control. **B.** Detection of protein expression of Nrf-2, GPx1 and Keap1 by Western blot. **C.** GPx1 and GR activities in GSCs. *P < 0.01 vs control. Error bars indicate the standard error of the mean of three individual experiments. **D.** GSH status in GSCs. *P < 0.01 vs control. Error bars indicate the standard error of the mean of three individual experiments.

We used lentiviral transduction to overexpress miR-153 (Figure 4A) and evaluate its effects in GPx1 expression and activity in GSCs. As shown in Figure 4B, Nrf-2 and GPx1 protein expression were significantly decreased in GSCs with stable integration of miR-153 compared with that of control cells. Keap1 expression showed no obvious changes in all tested cells. We further investigated the enzymatic activities of GPx1 and GR. A functional GR is essential as it allows the swift reduction of the oxidized GSSG, formed upon reduction of peroxides by GPx1, into the reduced GSH that serves as an electron donor to GPx1 [21, 22]. As shown in Figure 4C, GPx1 and GR activities were significantly decreased in GSCs with stable integration of miR-153 compared with that of control cells. Therefore, we further investigated the status of the GSH system in GSCs. As shown in Figure 4D, GSH was significantly decreased, while GSSG was significantly increased in GSCs with miR-153 overexpression. As a result, the GSH/GSSG ratio, an indicator of the cellular health, was significantly lower in GSCs with miR-153 overexpression. These results demonstrated that miR-153 overexpression would indirectly impair the GSH system in GSCs through targeting Nrf-2.

### MiR-153 overexpression increased ROS production, apoptosis and radiosensitivity of GSCs

**Figure 5:**
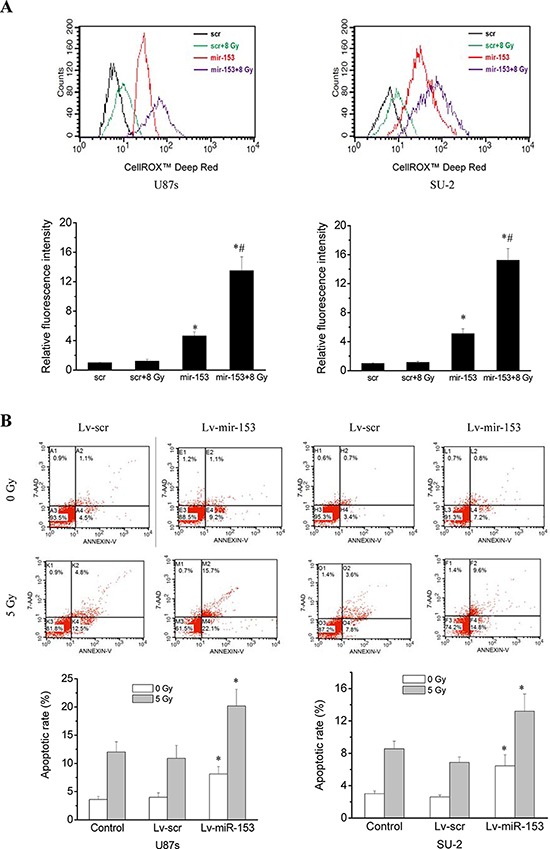

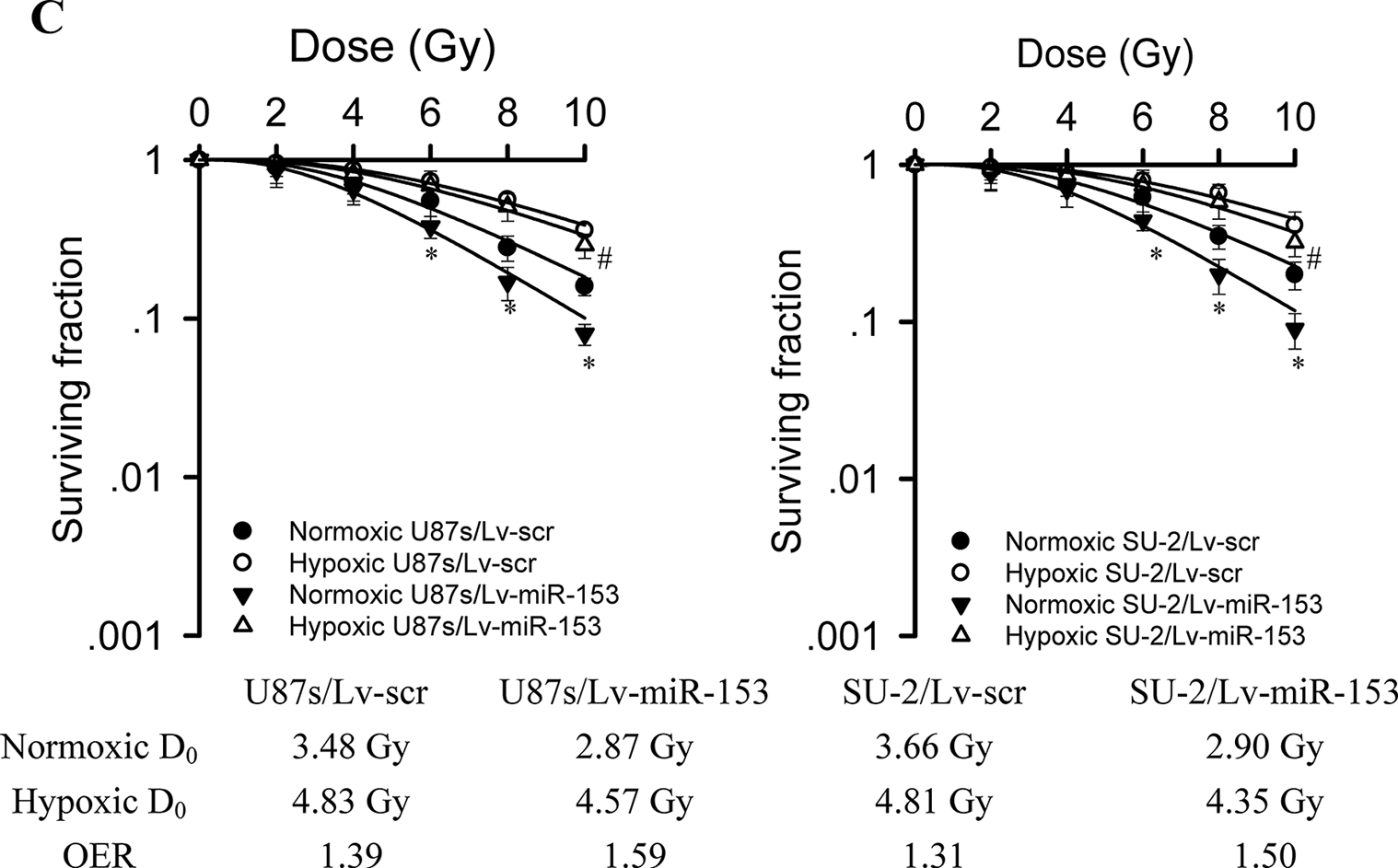
MiR-153 overexpression increased ROS production, apoptosis and radiosensitivity in GSCs **A.** Flow cytometric analysis of ROS formation in GSCs with miR-153 overexpression after exposure to ionizing radiation. *P < 0.01 vs scr. ^#^*P* < 0.01 vs miR-153. **B.** Apoptosis of GSCs were quantitatively measured using flow cytometry 48 h after 5 Gy X-ray irradiation. *P < 0.01 vs control. **C.** Survival curves of GSCs with miR-153 overexpression. After exposure to 0, 2, 4, 6, 8 and 10 Gy X-ray irradiation under normoxia or hypoxic conditions, cell survival fractions were examined and the survival curves of cells were obtained from data fitting according to the linear quadratic model. Error bars indicate the standard error of the mean of three individual experiments. *P < 0.01 vs normoxic U87s/Lv-scr or SU-2/Lv-scr. ^#^*P* < 0.05 vs hypoxic U87s/Lv-scr or SU-2/Lv-scr. OER (oxygen enhancement ratio) was calculated as the ratio of hypoxic D_0_ (mean lethal dose) to normoxic D_0_.

ROS generation was significantly increased in GSCs with miR-153 overexpression, whereas ROS generation in GSCs with stable integration of scramble sequence showed no obvious change 1 h after 8 Gy X-ray irradiation, as shown in Figure 5A. Apoptosis of GSCs were quantitatively measured using flow cytometry 48 h after 5 Gy X-ray irradiation. Our results showed that the average apoptotic rate of GSCs with miR-153 overexpression irradiated by 0 or 5 Gy X-ray were significantly increased compared with those of control cells (Figure 5B). The apoptosis of GSCs induced by miR-153 even in the absence of radiation might result from targeting B cell lymphoma 2 (Bcl-2) and myeloid cell leukemia sequence 1 (Mcl-1) genes, which has been reported by previous study [23]. After exposure to various dose of X-ray under normoxia or hypoxic conditions, the survival curves of cells, as shown in Figure 5C, were obtained. It is clear that GSCs with miR-153 overexpression were more radiosensitive than GSCs with stable integration of scramble sequence under normoxia or hypoxic conditions. Moreover, miR-153 overexpression resulted in a slightly higher OER in GSCs.

### MiR-153 overexpression decreased the neurosphere formation capacity and stemness of GSCs

**Figure 6:**
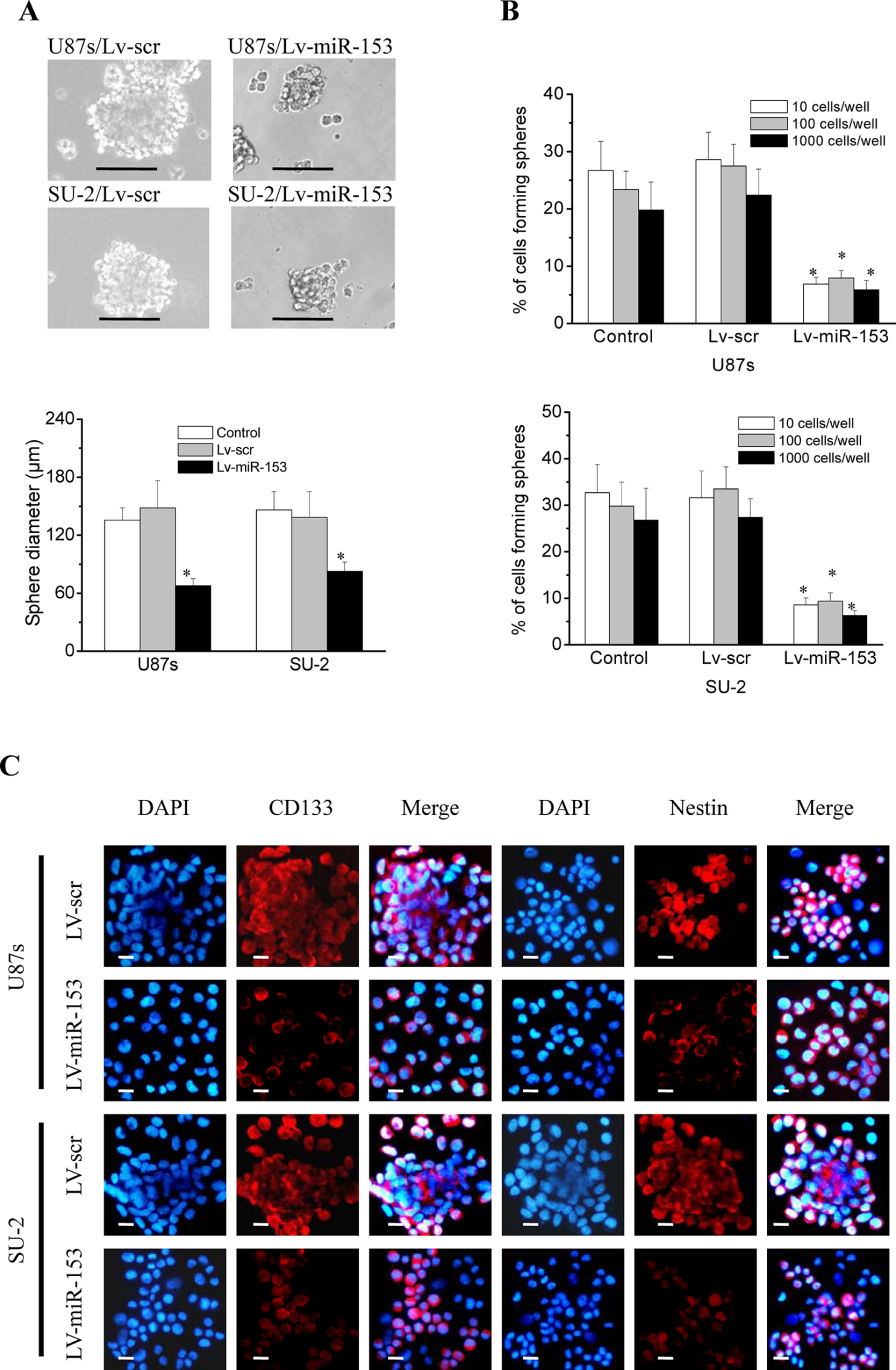

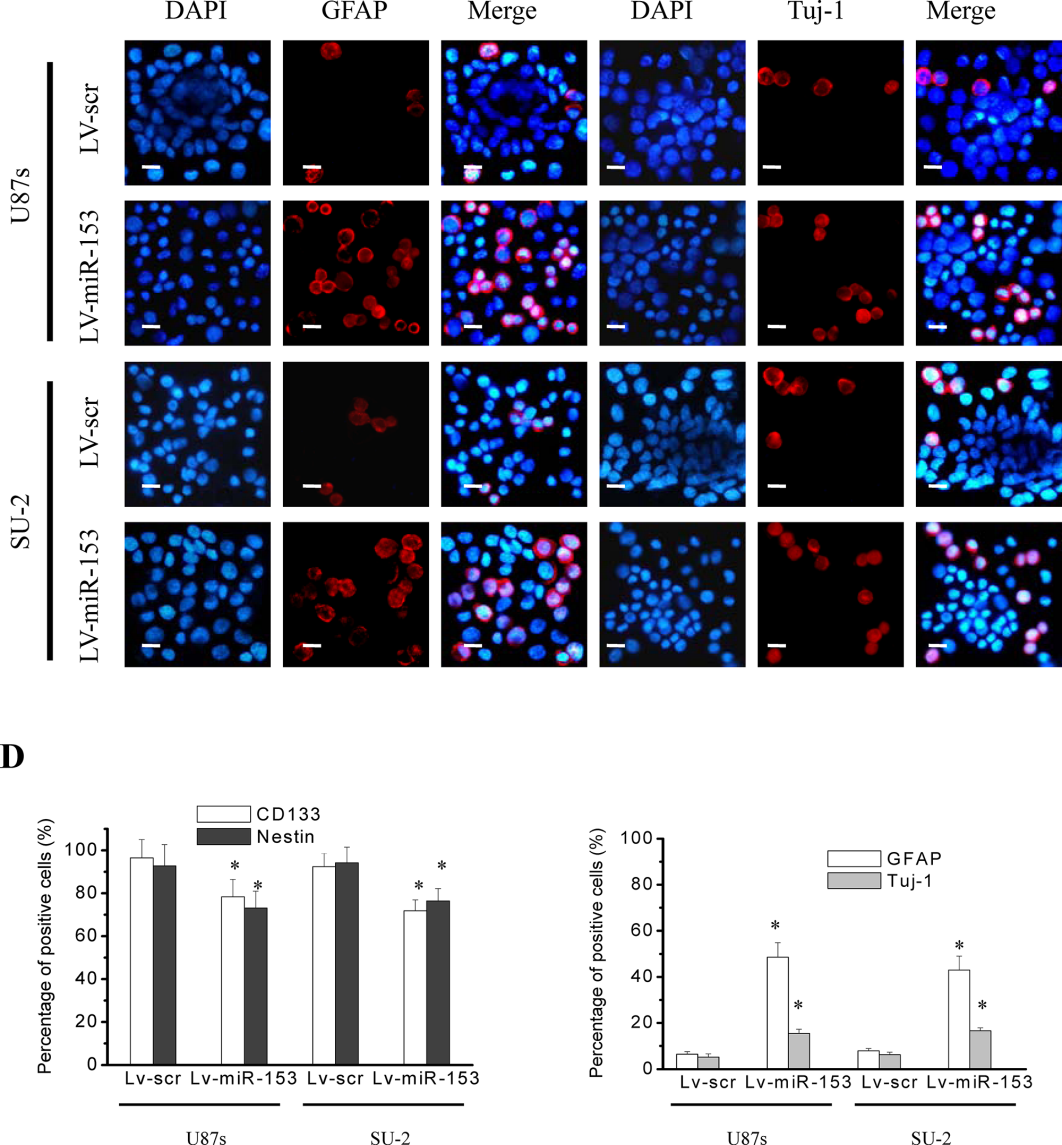
MiR-153 overexpression decreased stemness and induced differentiation in GSCs **A.** The diameters of neurospheres of GSCs with miR-153 overexpression plated at 100 cells per well in 96-well plates. *P < 0.01 vs control. Representative images of neurospheres (light microscope). Scale bar = 100 μm. **B.** The percentage of GSCs with miR-153 overexpression forming neurospheres. *P < 0.01 vs control. **C.** GSCs were immunostained with antibodies against the stem cell markers, CD133 and nestin, and the differentiation markers GFAP and Tuj-1. Fluorescence images were captured with fluorescence microscope. Scale bar =10 μm. **D.** The percentage of positive cells in GSCs. The percentage of positive cells was calculated as number of positive cells/total number of cells × 100 in 9 randomly selected fields (400 ×). *P < 0.01 vs Lv-scr cells.

GSCs were sorted into a 96-well plate at a density of 10, 100 or 1000 cells per well. After 12 d culture, the diameters of neurospheres of GSCs/Lv-miR-153 were significantly decreased compared with those of control cells (Figure 6A), and the percentage of cells forming neurospheres was also significantly decreased (Figure 6B). These results indicated that miR-153 overexpression decreased the neurosphere formation capacity of GSCs. GSCs were subjected to immunofluoresence staining for the stem cell markers CD133 and nestin and the differentiation markers glial fibrillary acidic protein (GFAP) (astrocytes) and Tuj-1 (neurons). Representative photomicrographs showed that miR-153 overexpression decreased CD133 and nestin immunostatining and increases GFAP and Tuj-1 immunostaining in both stem cell lines (Figure 6C, 6D). These results indicated that miR-153 overexpression decreased stemness and induced differentiation in GSCs.

### MiR-153 overexpression activated p38 MAPK signaling pathway via ROS in GSCs

**Figure 7:**
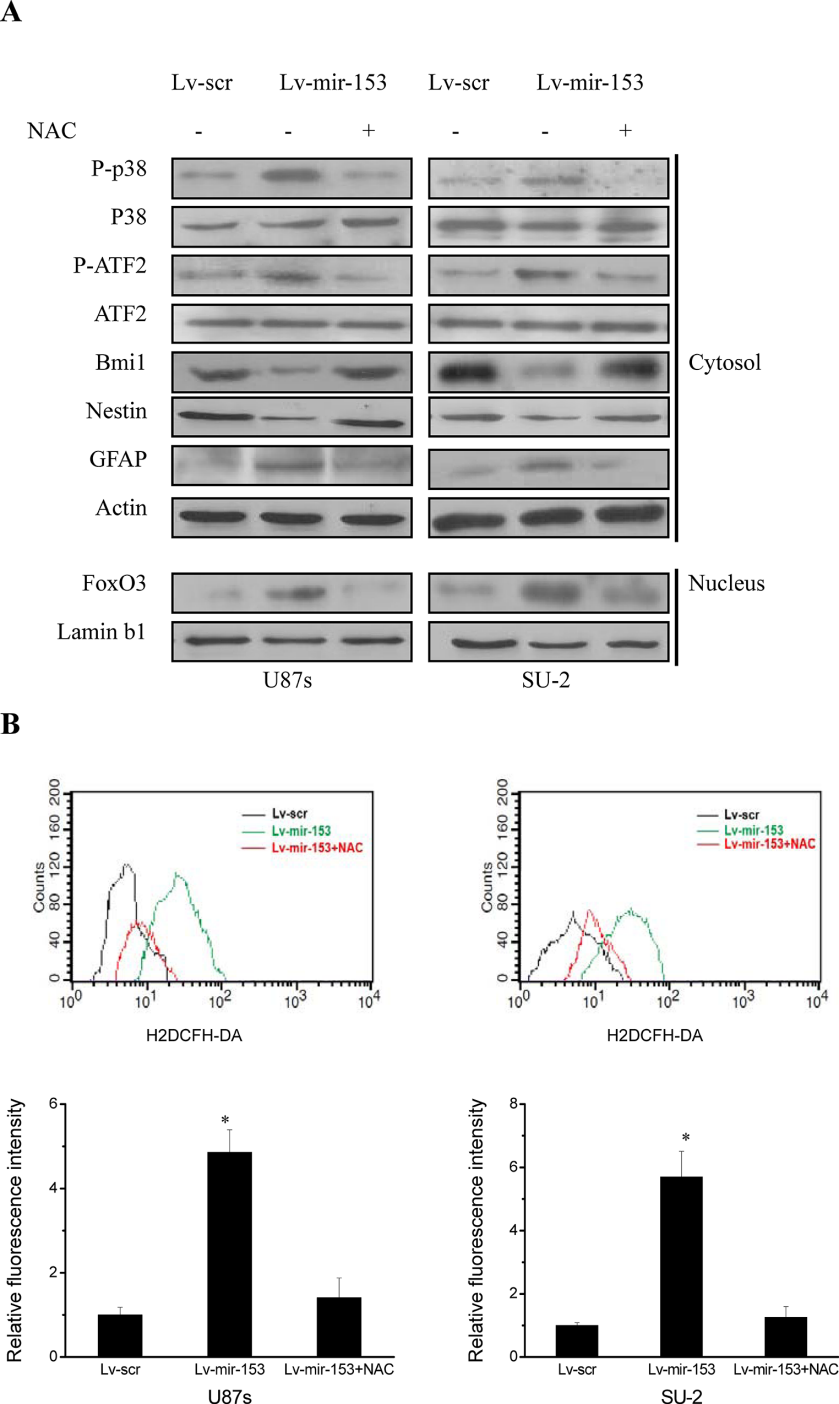
MiR-153 overexpression decreased stemness and induced differentiation through ROS-mediated activation of p38 MAPK in GSCs **A.** Detection of protein expression of p38 MAPK signaling pathway, stem cell markers and differentiation markers by Western blot. **B.** Flow cytometric analysis of ROS formation in GSCs with miR-153 overexpression in the presence and absence of an antioxidant N-acetylcysteine (NAC). *P < 0.01 vs Lv-scr.

It was reported that ROS-mediated activation of p38 mitogen-activated protein kinase (MAPK) plays a pivotal role in the control of differentiation and tumor-initiating capacity of GSCs, and ROS triggered p38-dependent Bmi1 protein degradation and FoxO3 activation in GSCs, which were responsible for the loss of self-renewal capacity and differentiation, respectively [24]. Thus, to identify the signaling pathways involved in miR-153 overexpression mediated differentiation of GSCs, we first determined whether p38 MAPK signaling pathway was activated. As shown in Figure 7A, miR-153 overexpression increased phosphorylated p38 and ATF2 and differentiation marker GFAP, and caused FoxO3 activation as evidenced by its accumulation in the nucleus. Meanwhile, the stem cell markers, Bmi1 and nestin, were decreased in GSCs with miR-153 overexpression. Moreover, N-acetylcysteine (NAC), which acts as a free radical scavenger by promoting intracellular biosynthesis of GSH, almost totally canceled the effect of p38 MAPK activation resulting from miR-153 overexpression. As shown in Figure 7B, miR-153 overexpression increased ROS formation in GSCs, which was abrogated in the presence of NAC. These results indicated that miR-153 overexpression decreased stemness and induced differentiation through ROS-mediated activation of p38 MAPK in GSCs.

### Nrf-2 overexpression rescued stemness and radioresistance of GSCs with miR-153 overexpression

**Figure 8:**
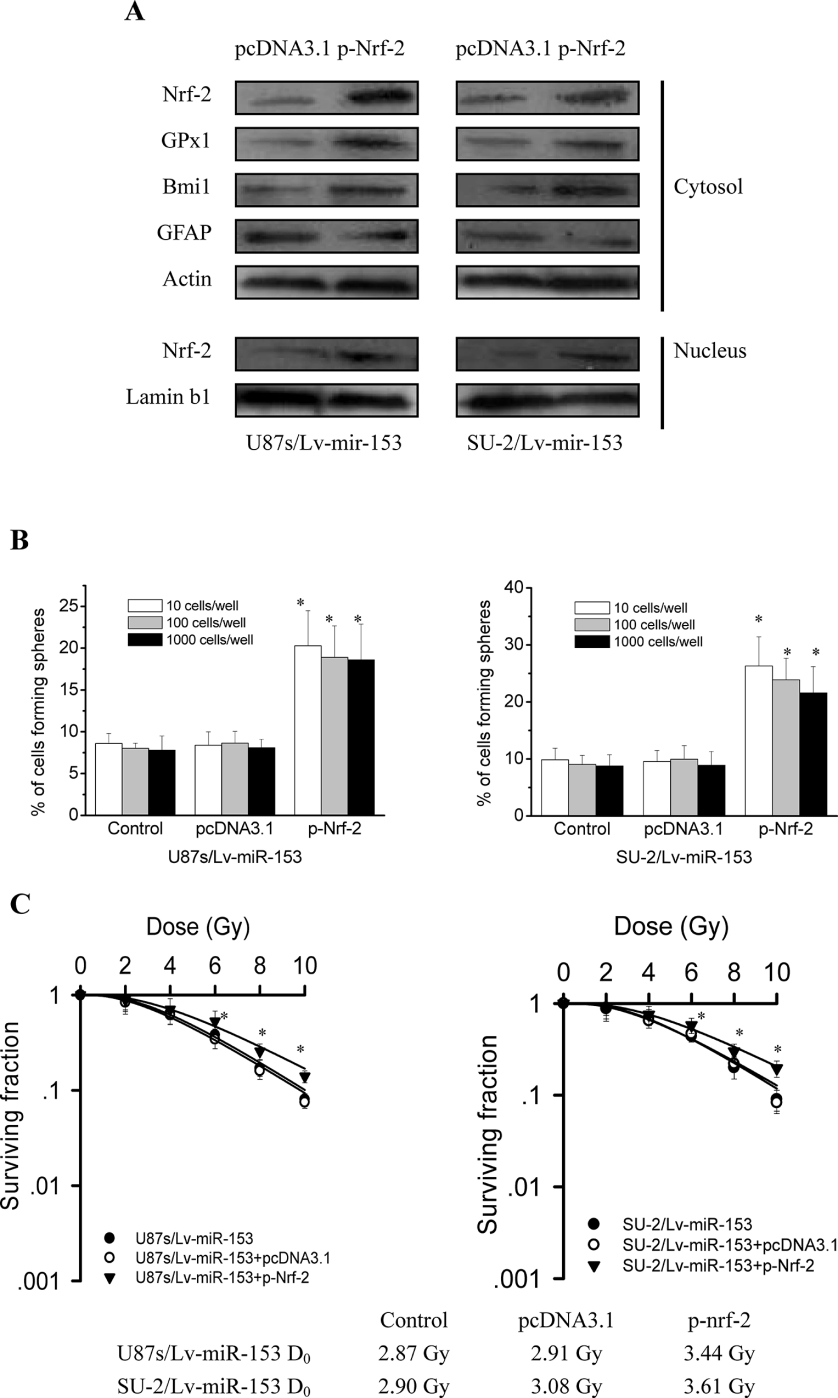
Nrf-2 overexpression rescued stemness and radioresistance of GSCs with miR-153 overexpression **A.** Detection of protein expression of Nrf-2, GPx1, stem cell marker Bmi1 and differentiation marker GFAP by Western blot. **B.** The number of neurospheres per well of GSCs/Lv-miR-153 transfected with Nrf-2 expression vectors. *P < 0.01 vs control. **C.** Survival curves of GSCs/Lv-miR-153 transfected with Nrf-2 expression vectors. After exposure to 0, 2, 4, 6, 8 and 10 Gy X-ray irradiation, cell survival fractions were examined and the survival curves of cells were obtained from data fitting according to the linear quadratic model. Error bars indicate the standard error of the mean of three individual experiments. *P < 0.01 vs U87s/Lv-miR-153 or SU-2/Lv-miR-153. D_0_ (mean lethal dose) was calculated by the linear quadratic model.

To investigate whether Nrf-2 overexpression could rescue stemness of GSCs with miR-153 overexpression, we detected the stem cell marker Bmi1 and differentiation marker GFAP expression by Western blot 48 h after Nrf-2 expression vectors transfection. As shown in Figure 8A, Nrf-2, GPx1 and Bmi1 protein expression were significantly increased, while GFAP protein expression were significantly decreased in GSCs transfected with p-Nrf-2 compared with those of GSCs transfected with pcDNA3.1. These results indicated that Nrf-2 overexpression could rescue the decreased stemness resulting from miR-153 overexpression in GSCs. As shown in Figure 8B, Nrf-2 overexpression also rescued the decreased neurosphere formation capacity of GSCs resulting from miR-153 overexpression. To investigate whether Nrf-2 overexpression could rescue radioresistance of GSCs with miR-153 overexpression, we examined radiosensitivity by clonogenic assay 48 h after Nrf-2 expression vectors transfection. As shown in Figure 8C, it is clear that GSCs transfected with p-Nrf-2 were more radioresistant than GSCs transfected with pcDNA3.1. These results indicated that Nrf-2 overexpression could rescue the decreased radioresistance resulting from miR-153 overexpression in GSCs.

### MiR-153 overexpression reduces tumorigenic capacity of GSCs

**Figure 9:**
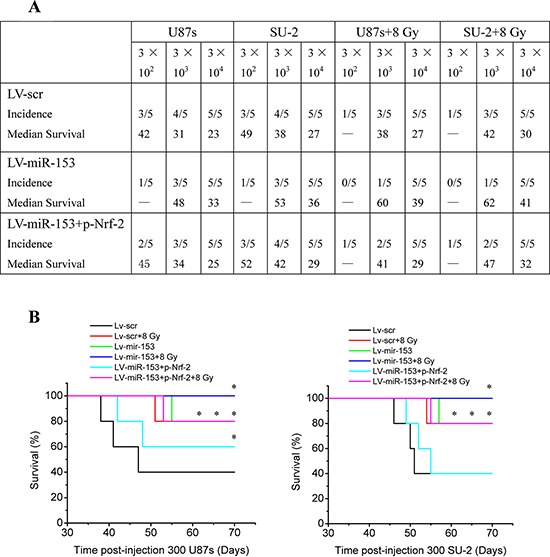

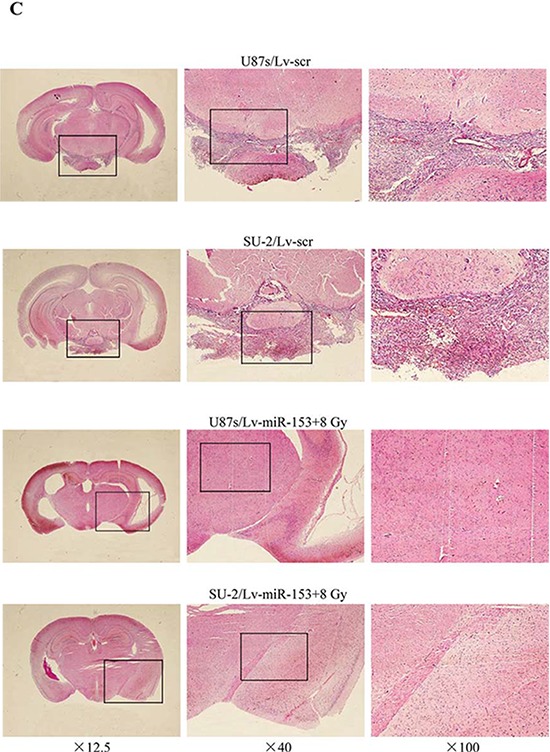
MiR-153 overexpression reduces tumorigenic capacity of GSCs **A.** Tumor incidence of GSCs and median survival of nude mice bearing human GSCs. **B.** Kaplan-Meier curves of mice bearing human GSCs. *P < 0.05 vs Lv-scr. **C.** Hematoxylin and eosin staining of brains of mice injected with human GSCs (*n* = 300).

Orthotopic transplantation is the gold standard for determining tumor-initiating potential of putative GSCs. To measure the effect of miR-153 overexpression on tumor formation, GSCs stably expressing miR-153 mature sequence or scramble sequence were intracranially implanted into immuno-compromised hosts. Tumor-bearing mice were monitored daily until the development of neurologic signs, including lethargy, ataxia, paralysis, or seizure, in each animal. When 300 or 3, 000 cells were injected, the tumor incidence of GSCs with miR-153 overexpression decreased compared with that of GSCs expressing scramble sequence. After transfection with p-Nrf-2, the tumor incidence of GSCs with miR-153 overexpression increased. In addition, the tumor incidence of GSCs decreased after 8 Gy X-ray irradiation (Figure 9A). For all cell numbers transplanted, the median survival of mice injected with GSCs was increased with miR-153 overexpression. Nevertheless, after transfection with p-Nrf-2, the median survival of mice injected with GSCs with miR-153 overexpression decreased. Kaplan-Meier curves further demonstrate significant increases in survival with introduction of miR-153 overexpression and/or 8 Gy X-ray irradiation (Figure 9B). Hematoxylin and eosin staining demonstrated the presence of brain tumors in mice injected with GSCs expressing scramble sequence, including the presence of glioma cells infiltrating normal brain. Gliomas were not observed in brains of mice injected with miR-153 overexpression GSCs irradiated by 8 Gy X-ray (Figure 9C). These *in vivo* data indicated that miR-153 overexpression could reduce tumorigenic capacity of GSCs and increase survival in mouse models of human glioma.

## DISCUSSION

The intracellular ROS are maintained at low levels by antioxidative enzyme systems [25–27], including SODs, catalase, GPx, and peroxiredoxin [28–30]. GPx enzymes exist in different isoforms and can be tissue-specific. GPx1 is the most abundant enzyme of GPx family, and present in all cells [31]. Overexpression of antioxidant enzymes in glioma cells can contribute to their resistance to some chemotherapeutic agents and radiotherapy [32]. For instance, overexpression of SOD, catalase, GPx, and GR in radioresistant clone of U251 glioma cell line leads to their resistance to cisplatin and radiotherapy [33]. In the present study, GSCs exhibited lower radiosensitivity and ROS levels than non-GSCs glioma cells, so we examined antioxidant enzymes expression and activity in GSCs and non-GSCs glioma cells. We found that non-GSCs glioma cells displayed significantly lower basal GPx1 expression and activity than GSCs. Moreover, knockdown of GPx1 radiosensitized GSCs, which suggested GPx1 expression contribute to radioresistance of GSCs. Dokic et al. also demonstrated that glioma cell lines exhibited a deficiency in GPx1 expression and activity, which led to an accumulation of reactive oxygen/nitrogen species (ROS/RNS) and subsequent death of cells after induction of oxidative stress, while astrocytes and glioma stem cell lines expressed active GPx1 and resisted ROS/RNS-mediated cell death [21], which was consistent with our results.

Nrf-2, a bZip transcription factor, is also involved in the regulation of intracellular ROS [34]. Once activated in response to electrophilic and oxidative stress, Nrf-2 mediates the expression of a spectrum of cytoprotective genes [35]. Many cancers express gain-of-function Nrf-2 mutants or loss-of-function Keap1 mutations that lead to constitutive Nrf-2 activation [36, 37], which contributes to the resistance against oxidative stress, such as chemotherapeutic drugs and ionizing radiation [38]. However, the role of Nrf-2 in GSCs remains unclear. Our data showed that Nrf-2 protein expression was significantly increased in cytosol and nucleus of GSCs compared with that of non-GSCs glioma cells. Recently, Zhu et al. also demonstrated that Nrf-2 was significantly increased expressed in nucleus of CD133^+^ GSCs compared with CD133^−^ glioma cells analysised by immunofluoresence staining [39]. The mechanism behind the variable Nrf-2 levels that exist between the GSCs and non-GSCs glioma cells requires further investigation.

MiR-153 is thought to be one of the brain-enriched miRs, which play crucial roles not only in the development and function of the central nervous system (CNS), but also in the pathogenesis of CNS disease, such as Parkinson's disease (PD) and Alzheimer's disease (AD) [40, 41]. MiR-153 is significantly down-regulated in glioma compared with normal brain tissues and decreases cell proliferation and increases apoptosis by targeting B cell lymphoma 2 (Bcl-2) and myeloid cell leukemia sequence 1 (Mcl-1) genes in glioma cell lines [23]. Zhao et al. found that miR-153 was downregulated in CD133+ GSCs and miR-153 inhibited self-renewal ability of CD133+ GSCs and induced apoptosis [42]. However, little is known about the molecular mechanisms of miR-153 functions in GSCs. In this study, Nrf-2 was identified as a target gene of miR-153 in GSCs by bio-informatic analysis, real-time PCR, and luciferase reporter assay, so the downregulation of miR-153 contributed to the elevated Nrf-2 expression in GSCs, at least in part.

Some studies have reported that suppression of the Nrf-2-mediated antioxidant defense system sensitizes cancer cells to ionizing radiation and chemotherapeutic drugs [36, 38, 43]. We employed lentiviral-mediated gene transfer technique to overexpress miR-153 in order to target Nrf-2 and analyzed phenotypic changes in GSCs. We found that miR-153 overexpression impaired the GSH system, increased ROS production, apoptosis and radiosensitivity, decreased neurosphere formation capacity and stem cell marker expression, and induced differentiation in GSCs by targeting Nrf-2. To identify the signaling pathways involved in miR-153 overexpression mediated differentiation of GSCs, we determined whether p38 MAPK signaling pathway was activated. The MAPK pathways transduce signals leading to diverse cellular responses, including cell growth, differentiation, proliferation, apoptosis, and stress responses to environmental stimuli [44]. The extracellular signal regulated kinase 1/2 (ERK1/2) pathway typically transduces growth factor signals that result in cell differentiation or proliferation, while cytokines and stress signals activate the c-Jun N-terminal kinases (JNKs) and p38 MAPK pathways which lead to stress responses, growth arrest, or apoptosis [45]. Once activated, p38 phosphorylates a variety of substrates in the cytoplasm and nucleus to regulate gene expression, cell cycle and cell differentiation [46, 47]. Our results showed that miR-153 overexpression increased phosphorylated p38 and ATF2 and downstream differentiation markers in GSCs and these effects were almost totally reversed by a free radical scavenger NAC, suggesting miR-153 overexpression inducing GSCs differentiation through ROS-mediated activation of p38 MAPK pathway. To investigate whether Nrf-2 overexpression could rescue stemness and radioresistance of GSCs with miR-153 overexpression, we detected the stem cell marker expression and examined radiosensitivity of GSCs 48 h after Nrf-2 expression vectors transfection. The results indicated that Nrf-2 overexpression could rescue the decreased stemness and radioresistance resulting from miR-153 overexpression in GSCs. Our results thus far determined an important role for miR-153 in GSC stemness, survival, and radioresistance *in vitro*, but the ultimate goal of any cancer stem cell–directed therapy is to provide therapeutic benefit *in vivo*. We therefore evaluated the ability of miR-153 overexpression to increase the survival of immuno-compromised mice bearing intracranially implanted human GSCs. For initial experiments, we performed an *in vivo* limiting dilution assay with GSCs stably expressing miR-153 mature sequence or scramble sequence. The *in vivo* data showed that miR-153 overexpression could reduce tumor-initiating potential of GSCs and increase survival in mice bearing human GSCs.

Taken together, our study demonstrated that i) Nrf-2 was a target gene of miR-153 in GSCs, ii) miR-153 was down-regulated in GSCs, which led to radioresistance through up-regulation of Nrf-2 and GPx1, and iii) miR-153 overexpression decreased radioresistance and stemness of GSCs *in vitro* and *in vivo*. These findings suggest that miR-153/Nrf-2/GPx1 pathway play a important role in regulating radiosensitivity and stemness of GSCs via ROS and targeting the miR-153/Nrf-2/GPx1 axis could be a novel approach in development of therapeutic strategies against glioma.

## MATERIALS AND METHODS

### Cell culture

The human glioma cell lines U87 and SHG44 were purchased from the Type Culture Collection of the Chinese Academy of Sciences and cultured in DMEM/F12 medium (1:1, Hyclone) supplemented with 10% fetal bovine serum (FBS) (Invitrogen) in a humidified atmosphere containing 5% CO2 at 37°C. The human GSCs culture U87s, derived from glioma cell line U87, were enriched using serum-free clone formation method with stem cell medium, which has been previously described [48]. The patient-derived GSCs culture SU-2, isolated from a surgical specimen of a 52-year-old female patient with glioma, was kindly provided by Dr. Ting Sun in neurosurgery laboratory of the first affiliated hospital of Soochow University [49–51]. GSCs were cultured as spheres in serum-free DMEM/F12 supplemented with 2% B27 Neuro Mix (invitrogen), 20 ng/mL epidermal growth factor (EGF) and 10 ng/mL basic fibroblast growth factor (bFGF) (PeproTech). The cultures were fed every 3 days with one-third volume of fresh medium. Cell passaging was performed by dissociation of spheres using NeuroCult chemical dissociation kit (StemCell Technologies). Neurospheres of around 8–12 passages were used for this study.

### Hypoxia induction

Cells treated with hypoxia were exposed to a steady flow of low-oxygen gas mixture (1% O2, 5% CO2, 94% N2) in a modular incubator chamber (MiniGalaxy, RSBiotech, Irvine, Scotland).

### Clonogenic cell survival assay

Cells were irradiated with X-ray (6 MV, the dose rate was 200 cGy/min) by a PRIMUS accelerator (SIEMENS Medical Solutions, Erlangen, Germany) at room temperature. After irradiation, a specific number of cells (100 for cells irradiated with 0 or 2 Gy, 200 for 4 Gy and 2000 for 6, 8 and 10 Gy) was plated in petri dishes in triplicate for clonogenic assay. Then the cells were incubated for 11 days. Colonies were fixed by 37% formaldehyde solution and stained with crystal violet and colonies of more than 50 cells were counted. Furthermore, the cell survival fraction was counted out and the mean lethal dose (D_0_) was calculated by the linear quadratic model. The oxygen enhancement ratio (OER) was calculated as the ratio of D_0_ (hypoxia) to D_0_ (normoxia).

### Detection of ROS

Cells were incubated at 37°C in the dark with 20 μM of 2′7′-dichlorodihydrofluorescin diacetate (H2DCFH-DA) or 5 μM CellROX™ Deep Red reagent (Invitrogen) for 30 minutes. Cells were harvested via trypsinization and were washed to remove the dye prior to flow cytometric analysis (Beckman Coulter, CA, USA).

### Western blot analysis of protein expression

Western blotting was performed using standard procedures. The following primary antibodies were used: mouse monoclonal anti-Catalase, Manganese Superoxide Dismutase (MnSOD), Copper/Zinc Superoxide Dismutase (Cu/ZnSOD), GPx1, Nrf-2, Keap1, phospho-p38, p38, phospho-Activating transcription factor-2 (ATF-2), ATF-2, B-cell-specific Moloney murine leukemia virus insertion site 1 (Bmi1), Forkhead box O3 (FoxO3), Lamin b1 and β-actin (Santa Cruz Inc. California, USA). Experiments were repeated three times. The relative levels of protein expression were normalized against protein levels of an internal control gene, β-actin, performed in the same run.

### Subcellular fractionation

Cells were homogenized in hypotonic buffer (10 mM Tris-HCl, pH 7.8, 150 mM NaCl, and 1 mM EDTA) containing 0.1% (w/v) Triton X-100. The lysates were centrifuged at 3000 rpm for 10 min at 4°C. The pellet was re-suspended in hypotonic buffer and re-centrifuged and was used as the nuclear fraction. The supernatant fraction was re-centrifuged at 16, 000 rpm for 20 min at 4°C and used as the cytoplasmic fraction.

### Analysis of enzymatic activity of Catalase, MnSOD, GPx1 and Glutathione reductase (GR)

Catalase, MnSOD, GPx1 and GR enzymatic activities were analyzed using a Catalase Assay Kit, Cu/Zn-SOD and Mn-SOD Assay Kit with WST-8, Cellular Glutathione Peroxidase 1 Assay Kit, and a Glutathione Reductase Assay Kit (Beyotime Institute of Biotechnology, Jiangsu, China), respectively, following the manufacturer's instructions.

### Reduced and oxidized glutathione (GSH and GSSG) measurement

Reduced and oxidized glutathione were measured using a GSH and GSSG Assay Kit (Beyotime Institute of Biotechnology, Jiangsu, China) according to the manufacturer's instructions.

### GPx1-siRNA design and transfection

The cDNA sequence of GPx1 was obtained from GenBank (NM_000581). The siRNA target design tools from Ambion were used to design GPx1-siRNA. GPx1-siRNA was designed and synthesized as follows (Sangon Inc. Shanghai, China): sense: 5′-CCAUUGACAUCGAGCCUG ATT-3′, antisense: 5′-UCAGGCUCGAUGUCAAUGGTC-3′. GSCs were plated 24 h prior to transfection. Cells were transfected in 6-well plates by use of Lipofectamine RNAiMAX (Invitrogen, Carlsbad, CA, USA). GPx1-siRNA and negative control siRNA (nc-siRNA) were used at 100 nM final concentration.

### Real-time reverse transcription-polymerase chain reaction (RT-PCR)

For GPx1 mRNA expression were analyzed using the SYBR Green PCR Master Mix (Applied Biosystems) on a 7300 Real-Time PCR System (Applied Biosystems, Foster City, CA) using standard conditions as previously described [48]. Fold changes in mRNA expression were quantified with the 2^−ΔΔCT^ relative quantification method using β-actin as house keeping control. The primer sequences used were as follows: GPx1 forward, 5′-AAGGTACTACTTATCGAGA ATGTG-3′ and reverse, 5′-GTCAGGCTCGATGTCAATGGTCTG-3′. MiRNA detection by real-time analysis involved reverse transcription of cDNA using a small RNA specific stem-loop RT primer. Once specific cDNA was generated, individual miRNA expression was assessed by real-time PCR according to the TaqMan MicroRNA Assay. Results were normalized to small nuclear RNA U6 that served as control and the data were expressed as Log 2 fold change in respective miR/U6 snRNA levels.

### Immunoprecipitation analysis

Protein lysates were prepared from GSCs and corresponding glioma cells. Approximately 200 μL protein lysates were incubated at 4°C with 50 μL of Protein G/A beads (Miltenyi Biotec, Auburn, CA) and followed by additions of 10 μL of Keap1 antibody with end-to-end rotation overnight. The immunoprecipitates were then loaded onto columns and rinsed with lysis buffer. Preheated (95°C) 1 × sodium dodecyl sulfate (SDS) gel loading buffer was loaded onto the column matrix and was incubated at room temperature for 5 min. After discharging the supernatant, 50 μL of 1 × SDS gel loading buffer was added to the immunoprecipitates, and the supernatants were then collected and loaded into 12%–15% sodium dodecyl sulfate polyacrylamide gel electrophoresis (SDS-PAGE) for Western blot analysis.

### Luciferase reporter assay

The 3′UTR of Nrf-2 gene contains a putative miR-153-target site: 5′-CTTTATAAGTAATTCTA TGCAA-3′. The wild type and mutated 3′UTRs of Nrf-2 were amplified by PCR from cDNA of U87s cells and were ligated into the pGL3 basic luciferase expression vector (pGL3-Luc) (Promega, Madison, WI, USA) at the 3′-end of the luciferase coding sequence. The pGL3-Luc vector containing wild type and mutated 3′UTRs of Nrf-2 and the internal control vector pRL-TK (Promega) were co-transfected into U87s and SU-2 cells. Twenty four hours later, 20 nM of miR-153 oligos (5′-UUGCAUAGUCACAAAAGUGAUC-3′) or scramble oligos (5′-UUCUC CGAACGUGUCACGUTT-3′) (GenePharma Co. Shanghai, China) were transfected into U87s and SU-2 cells. Luciferase activity was measured 48 h after vectors transfection using Dual-luciferase Reporter Assay System (Promega) according to the manufacturer's instructions.

### Generation of stable cell lines

GSCs with stable integration of the miR-153 mature sequence or scramble sequence were generated through lentiviral-mediated gene transfer [48]. To generate the respective viruses, 293T cells were transfected with the lentiviral vector, pGLV-miR-153-GFP or pGLV-scr-GFP, and the packaging plasmid PG-P1-VSVG, PG-P2-REV and PG-P3-RRE according to standard protocols. The target GSCs were infected with viruses encoding either miR-153 or scramble sequence and selected using puromycin. After 3 weeks, single clones were analyzed for positive green fluorescent protein (GFP) signals. The positive clones were expanded for additional testing.

### Flow cytometric analysis of apoptosis

Quantification of apoptotic cells was performed according to the Annexin-V-PE/7-AAD Apoptosis Detection Kit manufacturer instructions (KeyGen Biotech. Nanjing, China). Analyses were performed by a flow cytometer (BD FACScan). Phycoerythrin (PE)-positive and 7-amino-actinomycin D (7-AAD) -negative cells were regarded as apoptotic cells.

### Neurosphere formation assay

GSCs were plated at 10, 100 or 1000 cells per well in 96-well plates. After culture for 12 d, the number of neurospheres that contained more than 20 cells in each well was determined, and neurosphere formation rate was calculated as the number of neurospheres/100 × 100%.

### Immunofluoresence staining for the stem cell markers and the differentiation markers

GSCs were fixed in 4% paraformaldehyde (Sigma-Aldrich) for 15 minutes at room temperature, permeabilized using 0.1% Triton-X100 (Sigma-Aldrich) for 20 min and blocked in 5% Bovine serum albumin (BSA) (Sigma-Aldrich) for 1 h at room temperature. Then the GSCs were immunostained with mouse antibodies against human CD133 (1:200, Santa Cruz), nestin (1:300, Santa Cruz), glial fibrillary acidic protein (GFAP, 1:500, Abcam) and Tuj-1 (1:500, Abcam) for 45 min in darkness. Subsequent visualization for the stem cell markers, CD133 and nestin, and the differentiation markers, GFAP (astrocytes) and Tuj-1 (neurons), were performed with Texas Red-conjugated anti-mouse IgG (1:1000, Vector Laboratories) for 30 min at room temperature and the nuclei were counterstained with 4′, 6′-diamidino-2-phenylindole (DAPI, 1:500, Santa Cruz). Fluorescence images were captured with fluorescence microscope (Olympus BX50).

### Nrf-2 expression vectors construction and transfection

Full-length cDNA of Nrf-2 was isolated from cDNA of U87s cells and was amplified through RT-PCR using specific forward 5′-CACCATGGGAATGGACTTGGAGCTGCC-3′ and reverse 5′-CTAGTTTTTCTTAACATCTGGCTTCTTAC-3′ primers. The amplified cDNA fragment was cloned into the pcDNA3.1(+) vectors. The recombinant vectors were confirmed by the digestion analysis of restriction endonuclease and inserted sequences were verified by DNA sequencing. Cells with stable integration of miR-153 were plated 24 h prior to transfection. Cells were transfected in 6-well plates by use of Lipofectamine 2000 (Invitrogen, Carlsbad, CA, USA).

### Orthotopic transplantation assays

For intracranial implantation, 36 h after irradiation with 8 Gy X-ray, GSCs/Lv-scr, GSCs/Lv-miR-153 and GSCs/Lv-miR-153+p-Nrf-2 cells were counted and the indicated numbers of GSCs were implanted into the right frontal lobes of 6–8-week-old female athymic nude mice (Experimental Animals Center of Shanghai Institute of Life Science, Shanghai, China). Mice were maintained until the development of neurological signs. At the development of neurological impairment, the mice were perfused with 4% Paraformaldehyde (PFA), and brains were removed and placed in 4% PFA. All the animal experiments were conducted in accordance with Guidelines for the Welfare of Animals in Experimental Neoplasia.

### Statistical analysis

All statistical parameters were calculated with GraphPad Prism 6.01 (GraphPad Software Inc.). Student's *t* test was used for most data analysis. For comparisons among more than two groups, One-way Analysis of Variance (ANOVA) followed by Bonferroni post-test was performed. Mice survival were evaluated by the Kaplan-Meier method and analyzed by the log-rank test. *P* < 0.05 was considered to be statistically different.
